# Whole genome and 16S rRNA dataset of *Pectobacterium carotovorum* strain 21TX0081 isolated from a symptomatic onion foliage in Texas

**DOI:** 10.1016/j.dib.2022.108823

**Published:** 2022-12-13

**Authors:** Bed Prakash Bhatta, Manzeal Khanal, Subas Malla

**Affiliations:** a Department of Horticultural Sciences, Texas A&M University, College Station, TX 77843, United States; b Texas A&M AgriLife Research and Extension Center, Uvalde, TX 78801, United States

**Keywords:** Soft rot pathogen, *Pectobacterium*, Onion, Genomics, 16S rRNA, Whole genome sequencing

## Abstract

Bacterial soft rot is an important disease in onion caused by *Pectobacterium carotovorum*, a Gram-negative bacterium belonging to the Pectobacteriaceae family. The soft rot disease may occur during the preharvest (mature bulbs) or postharvest (transit, storage) stage. We collected symptomatic onion plants from the field and isolated bacteria from the foliage and bulbs. We extracted genomic DNA from the bacterial strain 21TX0081 isolated from onion foliage, conducted a polymerase chain reaction (PCR) based on 16S ribosomal RNA (rRNA)-specific primers, and classified the strain to genus *Pectobacterium*. To ascertain the species, genome features, and novelty of the strain, we conducted whole genome sequencing and computed average nucleotide identity (ANI) and digital DNA-DNA hybridization (dDDH) values. We also annotated the genome of *P. carotovorum* strain 21TX0081 using Rapid Annotation using Subsystem Technology (RAST) server. The raw, untrimmed forward and reverse sequence reads of 16S rRNA, and whole genome annotation features have been deposited at Mendeley Data. The datasets generated from consensus 16S rRNA and whole genome sequence have been deposited in National Center for Biotechnology Information (NCBI) repository. They will be useful for future research on comparative genomics and developing tools for disease management in onion.

**Specifications Table**

**Table:** 

Subject	Agricultural Sciences
Specific subject area	Plant Pathology, Agricultural Microbiology, Horticulture
Type of data	Sequence files (ABI, FASTA, GZ formats) Table Image Chart Graph Figure
How the data were acquired	Datasets for 16S rRNA were generated from Sanger sequencing of polymerase chain reaction (PCR)-purified products. Datasets for the whole genome were acquired using Illumina's next-generation sequencing technology (NextSeq 2000). Data for annotated genome (subsystem features) were obtained from the RAST server.
Data format	Raw Filtered Analyzed
Description of data collection	Genomic DNA from the *P. carotovorum* strain 21TX0081 was used for both 16S rRNA-based PCR and whole genome sequencing. Trimmed forward and reverse orientation reads obtained from Sanger sequencing were de novo assembled to produce a consensus 16S rRNA sequence. Paired-end reads generated from Illumina sequencing were processed to obtain the contig-level whole genome assembly. The whole genome annotation provided subsystem features with their counts.
Data source location	Institution: Texas A&M AgriLife Research and Extension Center City/Town/Region: Uvalde, Texas Country: USA
Data accessibility	**Repository name:** Mendeley Data and National Center for Biotechnology Information (NCBI) *For Mendeley Data****:*** The raw forward and reverse orientation reads of 16S rRNA are stored at Mendeley Data repository under the folder ‘**Pectobacterium_carotovorum_21TX0081_16S_rRNA**’. The folder contains the following files: **21TX0081_F.ab1** (raw forward reads) **21TX0081_R.ab1** (raw reverse reads) The raw genome annotation file from the RAST server/SEED database is also stored at Mendeley data under the folder ‘**Pectobacterium_carotovorum_21TX0081_GenAnnot**’. The folder contains the file: **21TX0081_genome annotation_SEED_Viewer.xlsx** Data identification number: 10.17632/zw44bpc7d8.1 10.17632/7rthcw52kj.1 Direct URL to data: https://data.mendeley.com/datasets/zw44bpc7d8 https://data.mendeley.com/datasets/7rthcw52kj *For NCBI****:*** **Data identification number:** Datasets on consensus 16S rRNA sequence has been submitted to NCBI with **GenBank ID: OP297199 (**https://www.ncbi.nlm.nih.gov/nuccore/OP297199**)** Datasets and information on the whole genome can be obtained from: BioSample: SAMN29993934 BioProject: PRJNA862766 Assembly: GCA_024506455.1 GenBank: JANIEP000000000.1
	**Direct URL to data:** **16S rRNA:**https://www.ncbi.nlm.nih.gov/nuccore/OP297199 **BioSample:**https://www.ncbi.nlm.nih.gov/biosample/29993934 **BioProject:**https://www.ncbi.nlm.nih.gov/bioproject/862766 **Assembly:**https://www.ncbi.nlm.nih.gov/assembly/GCA_024506455.1 **GenBank:**https://www.ncbi.nlm.nih.gov/nuccore/JANIEP000000000.1/ **Contigs:**https://www.ncbi.nlm.nih.gov/Traces/wgs/JANIEP01?display=contigs

## Value of the Data

• There are limited genomic studies on pathogenic onion bacteria, and these datasets will help to accurately identify and report the occurrence of soft rot causing bacteria in onion at genus, species, and strain levels.
• Researchers working in *Pectobacterium* can benefit from the genomic resources (16S rRNA sequences, whole genome assembly, genome annotation) in conducting plant microbe interaction studies.
• These datasets can be used in pathogenomics studies and develop robust diagnostic assays for *P. carotovorum*.

## 1 Objective

The objective of these datasets is to provide the 16S rRNA and whole genome sequence information on a new strain (21TX0081) of the bacterial soft rot pathogen, *P. carotovorum*, isolated from a diseased onion foliage collected in Texas, USA. The annotation of the whole genome sequence of the strain 21TX0081 provides comprehensive information on subsystems features and may serve as a molecular basis for understanding the pathogenesis-related genes and pathways causing soft rot in onions.

## 2 Data Description

**Fig. 1:**
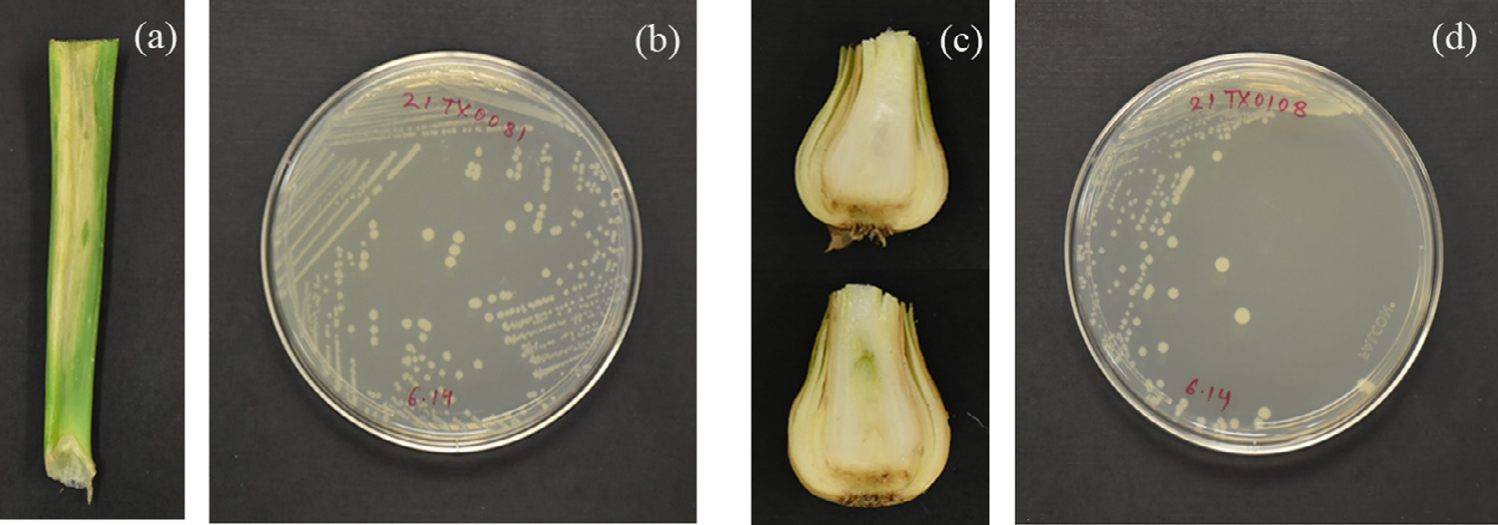
*(a)* Section of a diseased onion foliage collected from the field and used for isolation of bacterial strain 21TX0081, *(b)* Purified colonies of bacterial strain 21TX0081 in nutrient agar (NA) media, *(c)* Section of a diseased onion bulb collected from the field and used for the isolation of bacterial strain 21TX0108, and *(d)* Purified colonies of bacterial strain 21TX0108 in nutrient agar media.

Here we report the 16S rRNA and whole genome sequence of *P. carotovorum* strain 21TX0081 isolated from diseased onion foliage (Fig. 1a, 1b) collected from an onion field in Texas, USA. The consensus 16S rRNA sequence from this isolate is 1,382 base pairs (bp) long and has up to 99.78% match with *P. carotovorum*. The consensus 16S rRNA sequence had also 100% identity with the 16S rRNA sequence of *P. carotovorum* strain 21TX0108 (https://www.ncbi.nlm.nih.gov/nuccore/OP704110), isolated from a symptomatic bulb from the same field. Only two out of 20 samples (21TX0081 from foliage and 21TX0108 from bulb: Fig. 1c, 1d) or 10% of the total symptomatic samples collected from the field were classified as *P. carotovorum.* For isolate accessibility, a request should be made to the corresponding author.

The contig-level whole genome sequence of the strain has been deposited at NCBI (GenBank Assembly accession: GCA_024506455.1; https://www.ncbi.nlm.nih.gov/Traces/wgs/JANIEP01?display=contigs).

The folder ‘**Pectobacterium_carotovorum_21TX0081_16S_rRNA**’ in Mendeley Data contains files: **21TX0081_F.ab1** (raw 16S forward reads) and **21TX0081_R.ab1** (raw 16S reverse reads). Both files contain nucleotide sequences with their chromatograms showing quality scores. Usually, the bases on either end of the sequences have poor reads and must be trimmed prior to making a consensus sequence. These files can be visualized and end trimmed using various software such as SnapGene Viewer (https://www.snapgene.com/snapgene-viewer) and Geneious Prime (https://www.geneious.com/).

Several types of data for strain 21TX0081 have been deposited in NCBI. ‘**BioSample: SAMN29993934’** contains general information on the strain, such as the location of collection and isolation source. **‘BioProject: PRJNA862766’** contains general taxonomic information as well as the project data, including the assembly details. **‘Assembly: GCA_024506455.1′** comprises information on assembly method, genome coverage, sequencing technology, genome annotation data (e.g., number of genes, coding sequences, pseudogenes, etc.), and best-matching type-strain assembly. **‘GenBank: JANIEP000000000.1′** contains information on genome assembly and links to whole genome contigs – JANIEP010000001 to JANIEP010000047. The link for **‘Contigs’** data (https://www.ncbi.nlm.nih.gov/Traces/wgs/JANIEP01?display=contigs) includes a *‘Master’* tab with general genome information (number of contigs, proteins, genome length) and genome annotation data from the NCBI Prokaryotic Genome Annotation Pipeline (PGAP). The *‘Contigs’* tab lists 47 contigs with their names, lengths, proteins, and sequence files in different formats (e.g., FASTA, GenBank). The *‘Proteins’* tab has a list of 4,251 proteins with their accession number, length, product name, and sequence files in different formats (e.g., FASTA, GenPept). The *‘Download’* tab provides options to download the contigs and proteins in different file formats, and the *‘History’* tab shows the versions of the dataset.

The whole genome of bacterial strain 21TX0081 has 47 contigs with a genome size of 4.83 megabases (Mb). The contig-level genome annotated by NCBI Prokaryotic Genome Annotation Pipeline (PGAP) (https://www.ncbi.nlm.nih.gov/genome/annotation_prok/) [1] classified strain 21TX0081 to the species level: *P. carotovorum*. The best-matching type-strain for 21TX0081 (isolated from onion) is *P. carotovorum* strain NCPPB 312 (isolated from potato) (https://www.ncbi.nlm.nih.gov/assembly/GCF_000749855.1/). The genome features of both strains were derived from NCBI (Table 1). Average nucleotide identity (ANI) and digital DNA-DNA hybridization (dDDH) values between the two strains were 97.52% and 89.90%, respectively. The results indicated that the genome of strain 21TX0081 was not a novel species but a new strain of plant pathogenic *P. carotovorum*.

**Table 1:** Genome features of *Pectobacterium carotovorum* strain 21TX0081 compared with type-strain NCPPB 312; NCPPB = National Collection of Plant Pathogenic Bacteria, GC = guanine-cytosine content, rRNA = ribosomal RNA, tRNA = transfer RNA, NA = not available.

Genome features	*P. carotovorum* strain 21TX0081	*P. carotovorum* strain NCPPB 312
Genome Size (Mb)	4.83	4.76
GC%	51.95	51.91
Scaffolds or Contigs	47	55
Total number of genes	4,407	4,197
Total number of protein-coding genes	4,251	4,094
Total number of rRNAs	23	11
Total number of complete 16S rRNA	1	NA
Total number of tRNAs	73	69
Total number of pseudogenes	51	20

**Fig. 2:**
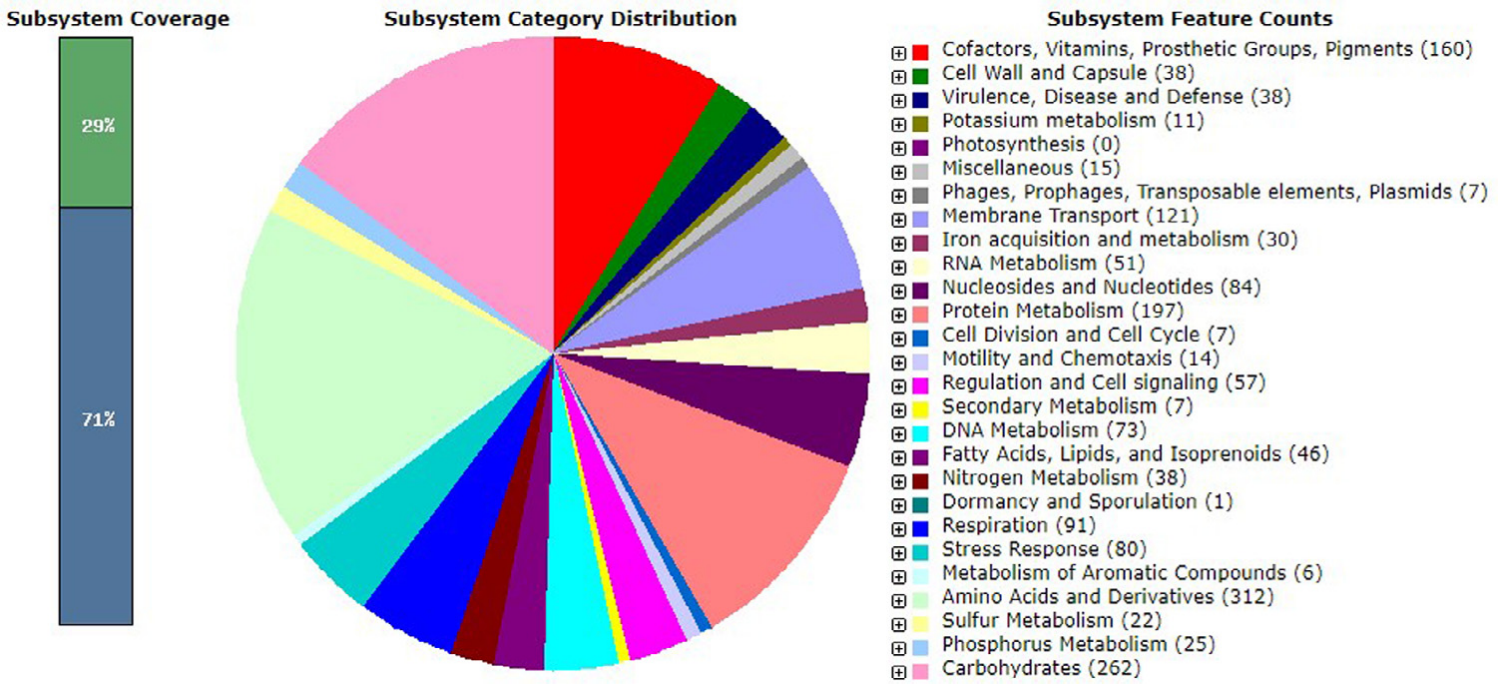
The pie-chart showing distribution of subsystem categories of *Pectobacterium carotovorum* strain 21TX0081 visualized in the SEED Viewer based on the annotation by the Rapid Annotation using Subsystem Technology (RAST) server. The bar diagram on the left indicates the percentage coverage of subsystems: green and blue bars indicate the percentage of total proteins encoded in the genome of strain 21TX0081 that could or could not be assigned to subsystem categories, respectively.

The genome annotation of *P. carotovorum* strain 21TX0081 using Rapid Annotation using Subsystem Technology (RAST) server resulted in a total of 4,573 coding sequences or protein encoding genes (pegs), of which 29% of the pegs in the genome of strain 21TX0081 could be assigned to 343 subsystems (Fig. 2). The folder ‘**Pectobacterium_carotovorum_21TX0081_GenAnnot’** in Mendeley Data contains the file: **21TX0081_genome annotation_SEED_Viewer.xlsx,** which provides information on category, subcategory, subsystem, role, and features of the pegs. Most of the subsystem features (annotated genes) were related to amino acids and derivatives (312 genes); followed by carbohydrates (262 genes); protein metabolism (197 genes); cofactors, vitamins, prosthetic groups, pigments (160 genes); and membrane transport (121 genes). Some interesting subsystems to further explore from the annotation include virulence, disease, and defense (38 genes), which contain genes related to adhesion, antibiotic resistance, and invasion/intracellular resistance. Further studies can also identify the interaction of genes in the sulfur metabolism subsystem (22 genes) with the bacterial strain and understand how the bacteria tolerate the organosulfur compounds in onion.

## 3 Experimental Design, Materials and Methods

### 3.1 Bacteria isolation, DNA extraction, and 16S based identification

Bacteria were isolated from ten symptomatic onion foliage and ten onion bulbs collected on May 10, 2021, from an overhead-irrigated onion field . Section of tissue (5 mm^2^) were cut around the margin of the symptomatic foliage/bulbs, rinsed with sterile water, macerated in 100 µL of sterile water, and streaked on a plate of nutrient agar (NA) using sterile loop [2,3]. The plates were incubated in the dark at 28 °C for 48 h. The predominant colonies were purified at least three times. DNA extraction was done from the purified bacterial colonies using DNeasy Power Soil Kit (Qiagen, MD, USA). Polymerase Chain Reaction (PCR) was done using the universal primers 27F (5′- AGAGTTTGATCMTGGCTCAG −3′) and 1492R (5′- CGGTTACCTTGTTACGACTT −3′) to determine the sequence of the 16S ribosomal RNA (rRNA) gene [4,5]. The PCR product was purified using the ExoSAP-IT Express kit (Applied Biosystems, Waltham, MA) following the manufacturer's instructions, and Sanger sequenced (Eurofins Genomics, USA). A consensus 16S rRNA sequence was made by trimming (error probability limit = 0.01) and combining (de novo assemble) forward and reverse reads in Geneious Prime 2021.1.1 (BioMatters, Inc., CA, USA) and searched for alignment using ‘*refseq_rna database’* in NCBI – Basic Local Alignment Search Tool (BLAST) [6]. The consensus sequence was also checked for the presence of chimeras via DECIPHER web tool (http://www2.decipher.codes/FindChimeras.html) [7] during sequence submission to NCBI GenBank.

### 3.2 Whole genome sequencing and de novo assembly

Genomic DNA was isolated from strain 21TX0081 and sent to SeqCenter (SeqCenter LLC, PA, USA) for whole genome sequencing. Sample libraries were prepared and sequenced on an Illumina NextSeq 2000 platform at an average of 100X sequencing depth, producing 2 × 150 bp paired-end reads. Demultiplexing, quality control, and adapter trimming of raw fastq files were performed with bcl-convert (v3.9.3) [8]. Paired reads in two different orientations (R1 and R2) were merged, and de novo assembled using Geneious Prime 2021.1.1 (BioMatters, Inc., CA, USA) [9].

### 3.3 Genome relatedness based on ANI and dDDH

The genome relatedness of the two strains was calculated using ANI based on the OrthoANIu algorithm (https://www.ezbiocloud.net/tools/ani) [10] and dDDH values obtained from Type (Strain) Genome Server-TYGS (https://tygs.dsmz.de/) [11,12]. The novelty of the species was determined based on the cutoff values: ANI values < 95∼96% or dDDH values < 70% for novel species and ANI values ≥ 95∼96% or dDDH values ≥ 70% for known species [13].

### 3.4 Genome annotation

Annotation of the genome of *P. carotovorum* strain 21TX0081 was done using the RAST server [14] (https://rast.nmpdr.org/) and SEED Viewer embedded in the SEED database [15] (https://www.theseed.org/wiki/Home_of_the_SEED).

## Ethics Statements

Not applicable. Research did not include human or animal subjects.

## CRediT authorship contribution statement

**Bed Prakash Bhatta:** Investigation, Methodology, Software, Data curation, Visualization, Writing – original draft. **Manzeal Khanal:** Investigation, Methodology, Software, Data curation. **Subas Malla:** Supervision, Conceptualization, Funding acquisition, Writing – review & editing.

## Declaration of Competing Interest

The authors declare that they have no known competing financial interests or personal relationships that could have appeared to influence the work reported in this paper.

## Data Availability

Pectobacterium_carotovorum_21TX0081_16S_rRNA (Original data) (Mendeley Data). Pectobacterium_carotovorum_21TX0081_GenAnnot (Original data) (Mendeley Data). whole genome (Original data) (NCBI).
